# Characteristics of Optic Disc and Visual Field Changes in Patients with Thyroid-Associated Orbitopathy and Open-Angle Glaucoma

**DOI:** 10.3390/jcm10173839

**Published:** 2021-08-27

**Authors:** Chih-Kang Hsu, Hsin-Yu Yao, Che-Min Lin, Hsu-Chieh Chang, Da-Wen Lu, Yi-Hao Chen, Ke-Hung Chien

**Affiliations:** 1 Department of Ophthalmology, Songshan Branch, Tri-Service General Hospital, Songshan 10581, Taiwan; chikanghsu@gmail.com; 2 Hau-Ming Eye Clinic Center, New Taipei City 22158, Taiwan; dokiki@pchome.com.tw; 3 Department of Ophthalmology, Taichung Armed Forces General Hospital, Taichung City 41152, Taiwan; cokacola0307@gmail.com; 4 Department of Nursing, Tri-Service General Hospital & National Defense Medical Center, Taipei 11490, Taiwan; n3197001@gmail.com; 5 Graduate Institute of Nursing, College of Nursing, Taipei Medical University, Taipei 11031, Taiwan; 6 Department of Ophthalmology, Tri-Service General Hospital & National Defense Medical Center, Taipei 11490, Taiwan; p310849@ms23.hinet.net (D.-W.L.); keane@ms18.url.com.tw (Y.-H.C.)

**Keywords:** hyperthyroidism, open-angle glaucoma, thyroid eye disease, thyroid-associated orbitopathy

## Abstract

This study aimed to characterize the changes in the visual field (VF) patterns and disc morphology of patients with thyroid-associated orbitopathy (TAO) and open-angle glaucoma (OAG). A retrospective review of the medical records at the Tri-Service General Hospital in Taiwan identified 396 eyes of 198 patients with thyroid-associated glaucoma. A final follow-up of VF examination in 140 eyes revealed 114 eyes with VF defects, indicating disease progression. The characteristics of and changes in disc morphology, optical coherence tomography findings, and VF defects were statistically analyzed. The most common VF defects at the initial diagnosis and the end of the follow-up period were inferior partial arcuate (17%) and paracentral (15%) defects, respectively. The most common VF defect in patients with unspecific disc signs was an unspecific scotoma (13%). The most common optic disc feature was disc cupping (51%), followed by parapapillary atrophy (48%). The most frequent location of nerve fiber layer thinning was the inferotemporal region (48%). VF defects showed a significantly more pronounced progression in the non-nerve fiber bundle group than in the nerve fiber bundle group (*p* < 0.001). This study details the characteristics and progression of disc morphology and VF defects in patients with TAO and OAG.

## 1. Introduction

Graves’ ophthalmopathy, also known as thyroid-associated orbitopathy (TAO) or thyroid eye disease, is an autoimmune inflammatory disease usually observed in dysthyroid patients [1]. Hyperthyroidism comprises the majority of TAO disease cases, with only a minority presenting a euthyroid or hypothyroid status. The incidence rate of TAO peaks between 30 and 50 years of age, with a sex distribution of sixteen women and three men per 100,000 population [1].

TAO pathogenesis is postulated as a series of effects caused by activating autoantibodies against the thyrotropin receptor and the insulin-like growth factor 1 receptor [2,3]. The consequences are the upregulation of fibroblast activity, accumulation of hyaluronidase-digestible material, and adipogenesis in the orbital tissue [4]. In clinical practice, the symptoms comprise eyelid lag, eyelid retraction, proptosis, extraocular muscle dysfunction, periorbital edema, scleral injection, exposure keratitis, and compressive optic neuropathy (CON).

Glaucoma is an optic neuropathy secondary to increased or normal intraocular pressure. To date, the relationship between TAO and glaucoma has been rarely investigated. Some researchers believe that edema and inflammation of the extraocular muscles, orbital adipose tissue, and connective tissue; accumulation of hyaluronic acid or mucopolysaccharide substances in the trabecular meshwork tissue; and an increase in episcleral venous pressure result in glaucoma [5]. According to previous studies, approximately 0.8–13.5% of patients with TAO develop open-angle glaucoma (OAG) [6,7,8]. However, studies regarding optic disc morphology and perimetric analysis in patients with TAO and glaucoma remains scarce.

Choi et al. focused on visual field (VF) changes in patients with TAO and CON in a recent study [9]. They collected 96 VF examination results from 68 patients and suggested a preliminary classification based on the location and shape of the VF defects. They found that an inferior VF defect was correlated with the presence of TAO and CON [9]. Freitag et al. reported sequences of VF progression in patients with CON [10]. Starks et al. reported their findings based on the correlation between orbital computed tomography and VF defect patterns [11]. However, a VF defect typical for patients with TAO and OAG has not been identified yet; therefore, this study aimed to characterize the VF patterns, disc morphology, and disc optical coherence tomography (OCT) patterns of TAO patients with OAG.

## 2. Materials and Methods

The medical records of patients with TAO at the Tri-Service General Hospital in Taiwan from 1 January 2006 to 31 December 2015 were reviewed. During the study period, patients aged 18 years or over with thyroid eye disease and glaucoma were eligible for inclusion in this study. TAO was diagnosed by oculoplastic specialists according to the NOSPECS criteria (Table 1), as reported by the American Thyroid Association [12]. OAG was diagnosed by two glaucoma specialists following gonioscopy, optic disc morphology, disc OCT, and VF examination. Exclusion criteria were as follows: (a) the presence of closed-angle glaucoma and secondary open-angle glaucoma (pseudoexfoliation type, pigment dispersion type and medication induced glaucoma), (b) TAO combined with other ophthalmologic diseases that may cause abnormal VF interpretation (including choroidal diseases, retinopathy, maculopathy, optic neuropathy, vitreous opacity, severe cataract, and corneal opacity), (c) high myopia (spherical equivalent less than −5 D), and (d) TAO with CON. All patients underwent a documented Humphrey VF test (24-2 or 30-2; Carl Zeiss Meditec, Dublin, CA, USA) during the study. The study was approved by the Ethics Committee of Tri-Service General Hospital (1-105-05-162) and performed according to the tenets of the Declaration of Helsinki. The requirement for informed consent was waived by the Ethics Committee due to the retrospective nature of the study.

VF examinations with reliability indices exceeding fixation losses >33% or false positive or false negative rates >33% were deemed unreliable and excluded from further analysis. The VF interpretation was considered abnormal in the following cases: (1) abnormal glaucoma hemifield test results, (2) pattern standard deviation depressed at the *p* < 5% level, and (3) a cluster of three non-edge points at an expected location for glaucoma, all of which were depressed on the pattern deviation plot at the 5% level and at least one of which was depressed at the 1% level. Specific VF defects were identified by two glaucoma specialists and categorized into subtypes according to the categories outlined in the Ocular Hypertension Treatment Study (OHTS) (altitudinal, superior or inferior arcuate, nasal step, paracentral, superior or inferior partial arcuate, temporal, wedge, central, hemianopia, inferior depression, partial hemianopia, partial peripheral rim, peripheral rim, quadrant, superior depression, total loss, vertical step, widespread, and unspecific) [13]. Additionally, we further categorized altitudinal scotoma, arcuate scotoma, and partial arcuate scotoma as superior, inferior, or superior and inferior. A different disc morphology was confirmed by two glaucoma specialists and classified. Disc morphology and disc OCT (Optovue, Fremont, CA, USA) data were also analyzed and classified as described below.

The data in the present study were analyzed using the SPSS software version 18.0 for Windows (SPSS Inc. Released 2009. PASW Statistics for Windows, Version 18.0. Chicago, IL, USA). All data are presented as the mean ± standard deviation. We conducted a Student’s *t*-test to compare the characteristics of the years of follow-up and mean deviation (MD) of different settings between groups. Values of *p* < 0.05 were considered statistically significant.

## 3. Results

According to the inclusion and exclusion criteria, 396 eyes of 198 patients were selected with initial diagnostic VF examinations. Of these, 314 VF examinations with identified defects were selected for further analysis. During the follow-up period (1 January 2006–31 December 2015), 140 eyes of 70 enrolled patients had final follow-up VF examinations, of which 114 VF examinations revealing defects were selected for further analysis. The follow-up periods ranged from 6 to 62 months (mean: 28.7 months). Detailed information of patients was provided in Supplemental Table S1.

### 3.1. Characteristics of VF Defects

**Figure 1:**
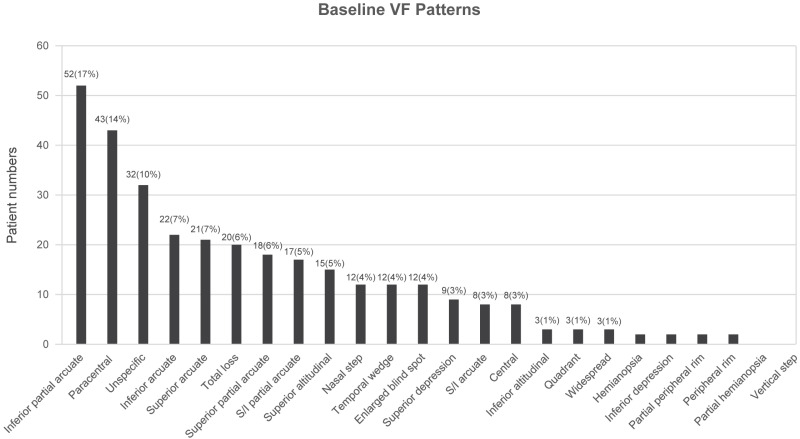
Distribution of visual field (VF) defects at initial diagnosis.

**Figure 2:**
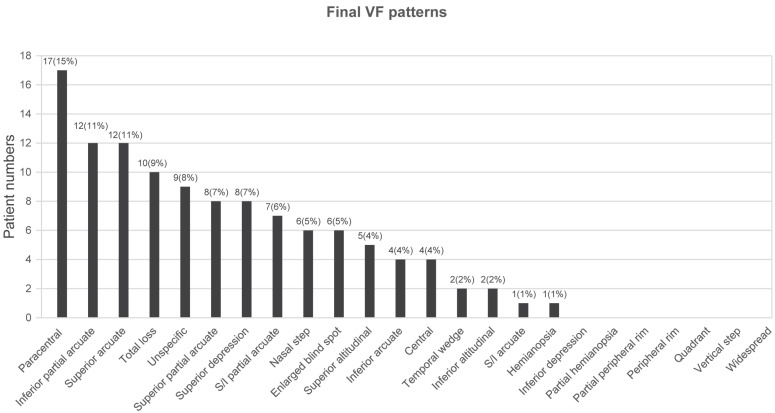
Distribution of VF defects at the end of the follow-up period.

**Figure 3:**
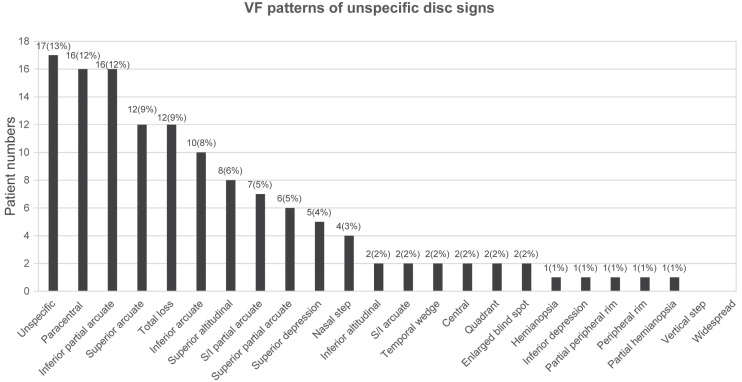
Distribution of VF defects in patients with unspecific signs.

Specific VF defect patterns were classified into subtypes according to the categories outlined in the OHTS [13] with further superior/inferior subcategories for altitudinal, arcuate, and partial arcuate scotomata as described in the Methods section. The ten most common VF defects at initial diagnosis were as follows: inferior partial arcuate (17%), paracentral (14%), unspecific (10%), inferior arcuate (7%), superior arcuate (7%), total loss (6%), superior partial arcuate (6%), superior and inferior partial arcuate (5%), superior altitudinal (5%), and nasal step scotoma (4%) (Figure 1). The ten most common VF defects at the end of the follow-up period were as follows: paracentral (15%), inferior partial arcuate (11%), superior arcuate (11%), total loss (9%), unspecific (8%), superior partial arcuate (7%), superior depression (7%), superior and inferior partial arcuate (6%), nasal step scotoma (5%), and enlarged blind spot scotoma (5%) (Figure 2). Additionally, VF patterns of patients with unspecific disc signs were analyzed, and the five most common VF defects in this population were as follows: unspecific scotoma (13%), paracentral scotoma (12%), inferior partial arcuate (12%), superior arcuate (9%), and total loss (9%) (Figure 3 and Table 2).

The initial VF defects and their incidence among enrolled patients included inferior partial arcuate (17%), paracentral (14%), unspecific (10%), inferior arcuate (7%), superior arcuate (7%), total loss (6%), superior partial arcuate (6%), superior and inferior partial arcuate (5%), superior altitudinal (5%), and nasal step scotoma (4%).

The VF defects and their incidence among enrolled patients at the end of the follow-up period included paracentral (15%), inferior partial arcuate (11%), superior arcuate (11%), total loss (9%), unspecific (8%), superior partial arcuate (7%), superior depression (7%), superior and inferior partial arcuate (6%), nasal step scotoma (5%), and enlarged blind spot scotoma (5%).

The VF patterns and the percentage of patients with unspecific disc signs included unspecific scotoma (13%), paracentral scotoma (12%), inferior partial arcuate (12%), superior arcuate (9%), and total loss (9%).

### 3.2. Characteristics of Disc Morphology and OCT

Disc morphology and disc OCT data from 396 eyes of the 198 enrolled patients were collected, analyzed, and classified (Table 3). The most common morphological feature of optic discs was disc cupping (50.75%), followed by parapapillary atrophy (PPA) (48.24%), unspecific disc signs (39.44%), lamina cribrosa dots (13.81%), and disc hemorrhage (3.01%). Of the 396 eyes investigated, 125 presented abnormal disc findings in the OCT. The part most commonly affected by nerve fiber layer (NFL) thinning was the inferotemporal region (48%), followed by the superotemporal (41.6%), inferonasal (28%), nasal-lower (24%), nasal-upper (24%), temporal-upper (19.2%), superonasal (18.4%), and temporal-lower (10.4%) areas.

### 3.3. MD Differences of VF

The MD differences between the initial diagnosis and the end of the follow-up period were analyzed in a total of 114 VF examinations (Table 4). The mean MD of the initial VF and the follow-up VF examinations were −4.42 ± 5.81 dB and −4.90 ± 6.86 dB, respectively. During the follow-up period, the mean MD difference between the initial diagnosis and the end of the follow-up period in the VF examinations was −0.19 ± 2.67 dB/year. The 114 VF examinations that had been performed both at the time of initial diagnosis and at the end of the follow-up period were further classified into two groups. If an initial VF examination revealed visual defects related to the nerve fiber bundle defect, the examined eyes were categorized into the group of nerve fiber bundle VF defects; otherwise, they were categorized as a non-nerve fiber bundle VF defect. Thus, we classified 97 VF and 17 VF examinations as nerve fiber bundle VF defects and non-nerve fiber bundle VF defects, respectively (Table 4). In the group of nerve fiber bundle VF defects, the mean MD of the initial and follow-up VF examinations was −3.99 ± 4.38 dB and −4.48 ± 5.83 dB, respectively. During the follow-up period, the mean MD difference between the initial diagnosis and the end of the follow-up period in the VF examinations was −0.15 ± 2.86 dB/year. In the group of non-nerve fiber bundle VF defects, the mean MD of the initial and follow-up VF examinations was −7.02 ± 10.99 dB and −7.45 ± 11.22 dB, respectively. During the follow-up period, the mean MD difference between the initial diagnosis and the end of the follow-up period in VF examinations was −0.46 ± 1.26 dB/year. Statistical analysis revealed a significant difference in MD between the initial diagnostic VF examinations and the end of the follow-up period VF examination. During the follow-up period, a significant difference in MD of VF examinations was also noted between the initial diagnosis and the end of the follow-up between the groups with non-nerve fiber bundle VF defects and nerve fiber bundle VF defects (*p* < 0.001).

## 4. Discussion

In patients with TAO complicated by glaucomatous signs, it is difficult for clinicians to clearly differentiate between glaucomatous optic neuropathy and CON. This study focused on disc morphology and VF defects in patients with TAO and open-angle glaucoma and found that annual MD changes were significantly different between patients with non-nerve fiber bundle VF defects and those with nerve fiber bundle VF defects according to the VF defect patterns. The analysis of information on atypical glaucomatous VF defect patterns, disc morphology, and disc OCT thinning helped us to more clearly describe the open-angle glaucoma patterns in patients with TAO.

Keltner et al. developed the OHTS system with three independent readers analyzing 2509 abnormal VFs [14]. In this OHTS classification, there are 17 mutually exclusive categories of visual field patterns, which describe either nerve fiber bundle defects or non-nerve fiber bundle defects. In their study, the most frequent VF defects were associated with glaucoma (47.9%), with non-nerve bundle defects accounting for only 4.8% of defects. The distribution of VF defects in that study reflects participants with glaucoma and ocular hypertension at a relatively incipient stage. In this study, most VF defects were compatible with glaucomatous VF defects at the initial diagnosis and follow-up; however, the percentages of patients with a total loss of VF (6% at initial diagnosis and 9% at the end of the follow-up period) were higher than those observed in the OHTS. We believe that the majority of patients with glaucoma are initially diagnosed at moderate or advanced stages due to the relative lack of symptoms at the incipient stage. Our data showing a higher incidence of total VF loss at the initial diagnosis support this opinion.

Paracentral scotomata in the VF seemingly has a high incidence at different disease stages, with 14% at initial diagnosis and 15% at the end of the follow-up period, which makes it the second most common and most common VF defect, respectively. Although paracentral scotoma is supposedly a typical VF defect in glaucoma, we predominantly observed inferior paracentral scotoma when we further categorized the subtype of paracentral scotomata. Similar inferior VF defects were noted in a study by Choi et al. [9], which focused on the VF of patients with TAO and CON, raising the question of whether an overlap of glaucomatous optic neuropathy and CON exists. Choi et al. [9] classified the visual field defect patterns according to the definitions in the OHTS [13] and concluded that the inferior VF defect is a typical feature of TAO with CON. Similar VF defects have been reported in other studies. Trobe et al. demonstrated that the most common VF pattern is “central scotoma and an inferior arcuate depression,” accounting for 23/36 (63%) of the eyes investigated [15]. Similarly, Soares et al. reported that 143/291 (49%) of eyes had some variants of inferior VF defect [16]. These stereotypical “other” inferior VF changes could be defined as the “TED-CON pattern”: a central inferior VF defect consisting of at least three contiguous points depressed by 5 dB or more, up to but not crossing the horizontal meridian and not contiguous from the blind spot to the nasal meridian. In our study, 27/43 (63%) of eyes with paracentral scotoma presented with typical glaucomatous optic neuropathy. However, the remaining 16 eyes showed unspecific disc signs, and 11 (69%) of these eyes showed inferior paracentral VF defects. The presenting VF defects of these unspecific disc signs imply a diagnostic dilemma between glaucomatous optic neuropathy and CON. Otherwise, these eyes may coexist with incipient compressive and glaucomatous optic neuropathy. Furthermore, in the study by Choi et al., nearly half of the VF defects (46/96 VFs, 47%) were typical glaucomatous VF scotomas [9]. Because they used billing codes to enroll VF examinations and did not mention the disc pathology, it is futile to investigate the relationship between high incidence of typical glaucomatous VF defects in patients with CON and disc pathology in their study. In the clinical setting, it is difficult to differentiate glaucoma from CON using only VF examinations. The disc morphology still plays an important role in the diagnosis of various optic neuropathies, including glaucoma and CON.

In patients with TAO and CON, vision loss occurs insidiously in cases of congestive inflammatory orbitopathy. In this study, the most common optic disc feature was disc cupping, followed by PPA and unspecific disc signs. Although PPA is a common characteristic of myopic optic discs, no relationship between PPA and TAO has been reported [17,18]. Such a high incidence of PPA in patients with TAO with glaucoma may render it a clinical feature indicative of glaucoma in patients with TAO.

NFL thinning on disc OCT examination revealed results similar to those in a previous glaucoma study [19]. The most common location of NFL thinning was the inferotemporal part, followed by the superotemporal, inferonasal, nasal-lower, nasal-upper, temporal-upper, superonasal, and temporal-lower regions. However, in patients with unspecific disc signs, there was a high proportion (53.5%) of patients with general advanced NFL loss at the first VF examination. Notably, atypical glaucoma presentation in patients with unspecific disc signs, advanced VF loss, and general NFL thinning on disc OCT examinations may require more clinical vigilance.

MD decline is an important VF parameter that implies the progression of glaucoma. In the Early Manifest Glaucoma Trial, the authors described that 122 untreated subjects with OAG presented with an MD decline of −0.6 ± 0.8 dB/year [20]. In addition, the Advanced Glaucoma Intervention Study found a mean worsening rate in treated OAG of −0.2 dB/year [21]. In our group of patients with nerve fiber bundle VF defects, the mean MD difference between initial diagnosis and the end of the follow-up in all VF examinations was −0.15 ± 2.86 dB/year during the follow-up period, which was significantly different from the corresponding value of −0.46 ± 1.26 dB/year in the group with non-nerve fiber bundle VF defects. VF defects in nerve fiber bundles were considered as typical glaucomatous changes, and the progression rates in the nerve fiber bundle group were comparable with those in the Advanced Glaucoma Intervention Study. Because non-nerve fiber bundle VF defects represent advanced optic damages, which may be the consequence of advanced glaucoma, a worse initial MD and a pronounced MD decline per year might have been noted in the non-nerve fiber bundle group. It is of clinical importance that although patients with TAO with glaucoma and advanced optic damage underwent adequate anti-glaucoma treatment, VF still progressed more in these patients than in the general glaucoma population.

This study had some limitations. First, a high percentage of patients in this study did not undergo follow-up examinations. This may have caused the rapid deterioration of VF defects and eventually VF loss in the analyzed patient group. Second, the VF analysis is also limited by the high incidence of unspecific VF defects, which may be the result of early and advanced VF losses or high comorbidity with ocular surface diseases, such as dry eye or exposure keratopathy in our patients. Third, in our analysis, we enrolled patients with thyroid eye disease and glaucoma with well-controlled intraocular pressure; however, visual prognosis in patients receiving orbital decompression was not mentioned; hence, this will be studied and reported in future studies.

## 5. Conclusions

This study provides a clinical spectrum of disc morphology, disc OCT, and VF characteristics in patients with TAO with glaucoma. Inferior paracentral VF defects may result from incipient compressive and glaucomatous optic neuropathy from an early stage. Since specific VF patterns may overlap in patients with CON and glaucoma, our report provides Supplemental Information for clinical ophthalmologists to differentiate between these two diseases. Further, more detailed information may be elucidated by future studies.

## Figures and Tables

**Table 1:** NOSPECS criteria according to the American Thyroid Association.

Class Signs
0 No symptoms or signs 1 Only signs; no symptoms (lid retraction and stare) 2 Soft tissue involvement (conjunctival and caruncle injection and chemosis; eyelid erythema, edema, and fullness) 3 Proptosis 4 Extraocular muscle involvement 5 Corneal involvement 6 Sight loss (optic nerve involvement)

NOSPECS criteria were used to evaluate the severity of thyroid-associated orbitopathy.

**Table 2:** Visual field (VF) examination patterns.

	Baseline VF	End of Follow-Up VF
**Number of eyes**	314	114
**Visual field pattern**		
**Nerve fiber bundle abnormalities**	223	76
Superior altitudinal	15	5
Inferior altitudinal	3	2
Superior arcuate	21	12
Inferior arcuate	22	4
S/I arcuate	8	1
Nasal step	12	6
Paracentral (inferior/superior)	43 (28/15)	17 (15/2)
Superior partial arcuate	18	8
Inferior partial arcuate	52	12
S/I partial arcuate	17	7
Temporal wedge	12	2
**Non-nerve fiber bundle abnormalities**	91	38
Central	8	4
Hemianopsia	1	1
Superior depression	9	8
Inferior depression	1	0
Partial hemianopsia	0	0
Partial peripheral rim	1	0
Peripheral rim	1	0
Quadrant	3	0
Total loss	20	10
Vertical step	0	0
Widespread	3	0
Enlarged blind spot	12	6
Unspecific	32	9

Values are presented as the number of patients. S/I, superior and inferior; VF, visual field.

**Table 3:** Optic disc morphology and disc optical coherence tomography (OCT) patterns.

	Number of Eyes (398 in Total)	Incidence
**Optic disc morphology**		
Cupping	202	50.75%
Disc hemorrhage	12	3.01%
PPA	192	48.24%
Lamina cribrosa dots	55	13.81%
Unspecific disc signs	157	39.44%
**Disc OCT**	Eyes with positive findings (125 in total)	
SN thinning	23	18.40%
ST thinning	52	41.60%
NU thinning	30	24.00%
TU thinning	24	19.20%
NL thinning	30	24.00%
TL thinning	13	10.40%
IN thinning	35	28.00%
IT thinning	60	48.00%

IN, inferonasal; IT, inferotemporal; NL, nasolateral; NU, nasal-upper; OCT, optical coherence tomography; PPA, parapapillary atrophy; SN, superonasal; ST, superotemporal; TL, temporolateral; TU, temporal–upper.

**Table 4:** Mean deviation (MD) differences in follow-up visual field (VF) examinations.

	All Study Participants	NFL Bundle Defect Group	Non-NFL Bundle Defect Group	*p*-Value *
Total VFs	114	97	17	
Follow-up years	2.32 ± 1.15	2.41 ± 1.14	1.76 ± 1.10	0.287
Initial VF MD	−4.42 ± 5.81	−3.99 ± 4.38	−7.02 ± 10.99	<0.001
End VF MD	−4.90 ± 6.86	−4.48 ± 5.83	−7.45 ± 11.22	<0.001
MD difference	−0.49 ± 3.11	−0.50 ± 3.32	−0.43 ± 1.22	0.144
Annual MD difference	−0.19 ± 2.67	−0.15 ± 2.86	−0.46 ± 1.26	<0.001

MD, mean deviation; NFL, nerve fiber layer; VF, visual field. * Student’s *t*-test.

## Data Availability

The datasets used and analyzed during the current study are available from the corresponding author on reasonable request.

## Supplementary Materials

The following are available online at https://www.mdpi.com/article/10.3390/jcm10173839/s1, Table S1: Background of patients in the study.
