# Supplementary material for: Characteristics of Optic Disc and Visual Field Changes in Patients with Thyroid-Associated Orbitopathy and Open-Angle Glaucoma
Source: J Clin Med. 2021 Aug 27;10(17):3839. doi: 10.3390/jcm10173839 (PMC8432097; doi:10.3390/jcm10173839)
Supplement: Supplementary file 1 [file jcm-10-03839-s001.zip › jcm-1332542-supplementary.pdf]

Table S1. Background of patients in the study

Parameter	Patients
CAS score at first visit (mean) (SD)	5.7 (2.1)
Minimum score (N) (%)	0 (27) (13.6%)
Maximum score (N) (%)	7 (36) (18.2%)
History of corticosteroid usage (N) (%)	161 (81.3%)
Oral route (N) (%)	124 (62.6%)
Intravenous route (N) (%)	73 (36.9%)
Prior radioactive iodine usage (N) (%)	21 (10.6%)
Prior radiotherapy (N) (%)	3 (1.5%)
Steroid responder (N) (%)	7 (3.5%)
Classes of glaucoma medical treatment (mean) (SD)	3.1 (1.6)
History of trabeculectomy surgery (N) (%)	26 (13.1%)
History of tube shunt surgery (N) (%)	0 (0)

CAS = clinical activity score. N=case number. SD = standard deviation.
